# Proteomics of Colorectal Cancer: Tumors, Organoids, and Cell Cultures—A Minireview

**DOI:** 10.3389/fmolb.2020.604492

**Published:** 2020-12-10

**Authors:** Philip H. Lindhorst, Amanda B. Hummon

**Affiliations:** ^1^Department of Chemistry and Biochemistry, The Ohio State University, Columbus, OH, United States; ^2^The Comprehensive Cancer Center, The Ohio State University, Columbus, OH, United States

**Keywords:** colorectal cancer, proteomics, mass spectrometry, organoids, cell culture, tumors, biomarkers

## Abstract

Proteomics, the study of the complete protein composition of a sample, is an important field for cancer research. Changes in the proteome can serve as a biomarker of cancer or lead to the development of a targeted therapy. This minireview will focus on mass spectrometry-based proteomics studies applied specifically to colorectal cancer, particularly the variety of cancer model systems used, including tumor samples, two-dimensional (2D) and three-dimensional (3D) cell cultures such as spheroids and organoids. A thorough discussion of the application of these systems will accompany the review of the literature, as each provides distinct advantages and disadvantages for colorectal cancer research. Finally, we provide conclusions and future perspectives for the application of these model systems to cancer research as a whole.

## Introduction

Colorectal cancer is the third-most prevalent, and second-most deadly, cancer worldwide (Global Cancer Observatory, 2018). In the United States, it is estimated there will be 147,950 new cases of colorectal cancer and will claim over 50,000 lives in 2020 alone (American Cancer Society, 2020). Early diagnosis and various medical treatments can reduce the fatality rate, but it requires further understanding the intricacies of cancer formation, survival, and spread at the molecular level. As seen in a 2010 review, the field of omics has tremendous potential for new molecular discoveries that change the way we treat colorectal cancer (Nambiar et al., 2010). Omics research analyzes cancer at the molecular level. For example, a genomics study of colorectal cancer patients may discover a recurring mutation that could be a factor in tumor formation, while a metabolomics study has the potential to discover a small molecule secreted into the bloodstream that could be used as a diagnostic biomarker for the presence of colorectal cancer.

**FIGURE 1:**
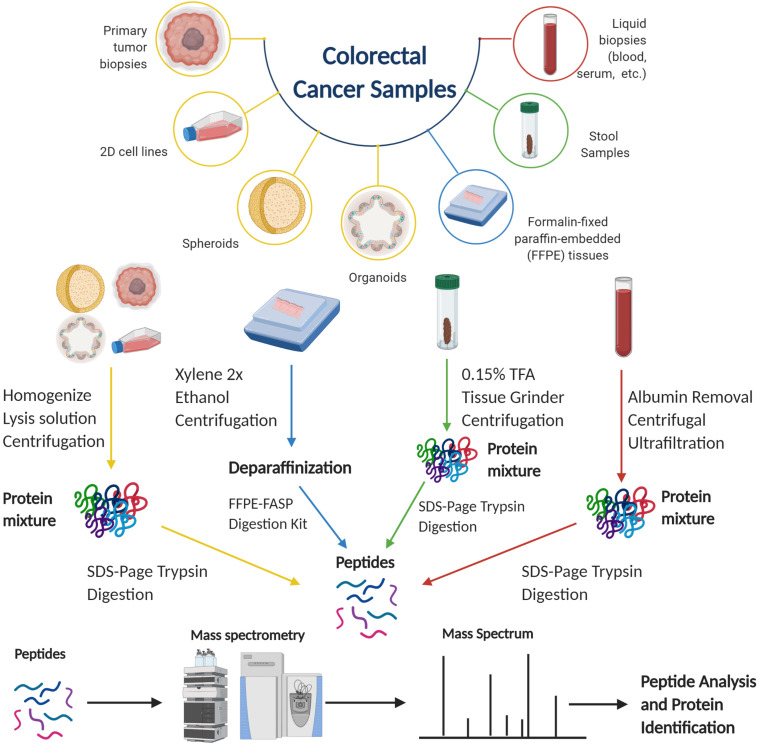
Proteomics workflows for different colorectal cancer samples (Tirumalai et al., 2003; Ang et al., 2010; Quesada-Calvo et al., 2017). Figure created with BioRender.com.

Proteomics is the study of proteins and can encompass the identification, quantification, localization, turnover, and regulation in a biological system. Liquid chromatography-mass spectrometry (LC-MS) has seen substantial use for proteomics in recent decades in which it has been utilized to obtain a global proteomic profile of a biological sample (Cravatt et al., 2007). In a mass spectrometry-based proteomics experiment, one method to obtain the identification and quantification of proteins is through bottom-up proteomics. As seen in Figure 1, proteins are extracted from a biological sample; the sample undergoes several steps to unfold all the proteins, reduce and alkylate the cysteine residues, before digestion into peptides by a proteolytic enzyme. The lysate undergoes chromatographic separation, typically by low-pH reversed-phase

chromatography before being analyzed by mass spectrometry. As peptides elute out of the column, the MS fragments the peptides to generate sequence-specific peaks. The methodology is outside the scope of this review; however, for a detailed explanation, Zhang et al. (2013) published an excellent overview of this approach.

Proteomics can be used in many facets of cancer research, beginning by being able to analyze the protein expressions in healthy and cancerous cells to identify differences in the protein abundance between the two populations. This information can be important to understand potential therapeutic targets in cancer cells. In addition to obtaining the basal proteome, mass spectrometry-based proteomics experiments can also be used to further evaluate the effect of a perturbation (adding a drug, knocking out a gene, etc.) by observing any proteomic alterations between control and treated groups. However, tumor heterogeneity is a major obstacle that has to be factored into any proteomics experiment due to the composition of cancer cells. Imperial et al. (2018) previously showed that the proteome throughout an individual tumor, as well between tumors at different locations, may be different. For a more detailed discussion of tumor heterogeneity, Lim and Lim (2018) provided an excellent review for solutions to overcoming this common problem. Additionally, it can be difficult to procure primary tumor samples and patient approval has to be obtained for their use. Due to these obstacles, it is often more favorable to use models that simulate the colorectal cancer proteome. With this in mind, researchers have developed several *in vitro* models, with varying degrees of complexity to study colorectal cancer.

This minireview highlights the use of mass spectrometry-based proteomics for primary colorectal tumor tissues studies and various models of colorectal cancer that are currently available. Since colorectal cancer proteomics is a rapidly evolving area of research, we will also provide our perspective on the future of this field in the new decade.

## *In vivo* Colorectal Cancer Proteomics

*In vivo* colorectal cancer samples, samples obtained from either a patient or mouse model, are the most desirable source of proteomic information and have been used for colorectal cancer research for a number of years. Patient-derived *in vivo* samples can include primary tumor tissue, formalin-fixed paraffin-embedded (FFPE) tissue, liquid biopsies (plasma, serum, etc.), and stool samples. Much of the literature from the last 20 years focuses on these samples and has been previously reviewed (Tjalsma, 2010; Lee et al., 2018; Martins et al., 2019). In this section, we will briefly focus on previous studies using specifically primary tumor tissue samples, then more thoroughly discuss why *in vitro* model systems are useful and seeing increased popularity in the last decade.

### Primary Tumors

For colorectal cancer research, no sample is more valuable than tissue directly from patients, as these samples provide the most accurate representation of the cancer’s proteome. Previously, the major focus of colorectal cancer proteomics research is the search for protein biomarkers for colorectal cancer, elucidated by comparing the proteomes of cancerous and non-cancerous tissue.

Protein biomarkers are proteins that are notably up- or down-regulated in the cancer proteome as compared to the normal proteome. Ideally, these proteins can serve as diagnostic markers of the presence of cancer, provide us with more information on cancer formation or survival, or possibly lead us to treatments that target these proteins. Using comparative proteomics with healthy and cancerous tissues, recent approaches show recognizable differences between these populations. The differences in protein abundance result in the identification of protein biomarkers, which are detailed in Table 1.

**TABLE 1:** Protein biomarkers of colorectal cancer using mass spectrometry.

**Model type**	**Protein**	**Up/down-regulated**	**Biomarker expression**	**References**
Primary tumor	Transgelin-2	Up-regulated	Correlation with lymph node metastasis	Zhang et al., 2010
Primary tumor	Matrix metalloproteinase-9	Up-regulated	Increased expression in both non-metastatic and metastatic tumor tissue	Saleem et al., 2019
Primary tumor	Caveolin-1	Down-regulated	Decreased expression in both non-metastatic and metastatic tumor tissue	Saleem et al., 2019
Primary tumor (FFPE)	OLFM4, KNG1. Sec24c	Up-regulated	Increased expression in early CRC stages, with decreasing expression in late stages (non-metastatic)	Quesada-Calvo et al., 2017
Primary tumor	ALDH1A1, OLFM4, HSPE1, SORD	Up-regulated	Significant expression in early CRC stages, but no change in expression in late stages (non-metastatic)	Besson et al., 2011
Primary tumor (FFPE)	Cyclophilin A, Annexin A2, Aldolase A	Up-regulated	Increased expression in colorectal cancer cells. Decreased secretion of aldolase A in human serum.	Yamamoto et al., 2016
Primary tumor	HSP47	Up-regulated	Increased number of HSP47-positive spindle cells in CRC stroma linked to lymph node metastasis	Mori et al., 2017
Primary tumor	COL12A1, CALU, BGN	Up-regulated	Increased expression along malignant progression from normal colon tissue to adenocarcinoma	Mikula et al., 2011
Primary tumor	MAOA, ENTPD5, MOSC2	Down-regulated	Decreased expression along malignant progression from normal colon tissue to adenocarcinoma	Mikula et al., 2011
2D cell culture/primary tumor	STMN1	Up-regulated	Highly increased expression in E1 cell line, the metastatic derivative of HCT-116 CRC cell line, and metastatic primary tumor tissues	Tan et al., 2012
2D cell culture/primary tumor	Cdc42BPA	Up-regulated	Significantly increased expression in highly invasive CRC cell lines and lymph node metastatic tumor microarrays	Hu et al., 2018
2D cell culture	Retinol-binding protein 1	Up-regulated	Increased expression in the HCT-116 CRC cell line as compared to normal derived colon cell line	Ludvigsen et al., 2020
2D cell culture	ERSP1	Up-regulated	Increased expression in CRC cells correlated with increased metastasis. Modulating its expression changed the expression of other cancer-related proteins	Ala et al., 2020
2D cell culture	SRSF3	Down-regulated	Decreased expression associated with CRC progression and increased metastasis	Torres et al., 2018
Organoid	MIF/CD74	Up-regulated	Increased expression in CRC organoids. Inhibition using a drug resulted in organoid disaggregation and death	Bozzi et al., 2017
Organoid	HMGCS2, CEMIP, LRP1, DPP4	Up-regulated	Increased expression in organoids where the removal of the APC gene activates an oncogenic Wnt response	Michels et al., 2019
Organoid	EPHA2, BCAM	Down-regulated	Decreased expression in organoids where the removal of the APC gene activates an oncogenic Wnt response	Michels et al., 2019

Examples of protein biomarkers include the protein OLFM4, found by two separate labs as a biomarker for colorectal cancer using bottom-up proteomics (Besson et al., 2011; Quesada-Calvo et al., 2017). Zhang et al. (2010) identified transgelin-2 as a biomarker of colorectal cancer, while Yamamoto et al. (2016) identified aldolase A. Mori et al. (2017) discovered shock protein 47 as a biomarker for the metastasis of colorectal cancer to lymph nodes. Mikula et al. (2011) used a combination of proteomics and transcriptomics to search for new protein biomarkers. Hao et al. (2017) used a novel algorithm to identify changes in abundance of entire protein pathways. They found elevated expression of proteins associated with chromatin modification and gene expression, but decreased expression of proteins responsible for core matrix architecture. These results would support the high mutation rate observed in cancer. Saleem et al. (2019) compared the proteomes of healthy tissue, non-adenomatous colon polyps, non-metastatic tumors, and metastatic tumors to identify proteins with elevated expression in the cancerous samples, such as matrix metalloproteinase-9, and decreased expression, such as caveolin-1. Similarly, Knol et al. (2014) compared benign and malignant tumor proteomes by enriching the protein fractions for chromatin-binding proteins, which had shown a difference previously (Albrethsen et al., 2010). Wisniewski et al. (2012) found that the proteome of colon adenocarcinoma tissue was significantly remodeled in comparison to normal tissue, which led them to compare the proteomes of colorectal mucosa, adenoma, and cancer (Wiśniewski et al., 2015). They noted significant changes in fatty acid metabolism and plasma membrane transporter proteins. New workflows using iTRAQ labeling (Jankova et al., 2011) or a centrifugal proteomic reactor (Liu et al., 2016) improve the data acquired from proteomic experiments. For further information on protein biomarkers, we suggest the following reviews: Tjalsma (2010); Lee et al. (2018), and Martins et al. (2019), which provide more information on recent advances and discoveries.

In contrast with the previously mentioned comparative studies, other cancer proteomics studies have utilized different approaches, for example, comparing the proteome with the genome or transcriptome. The relationship between the genome and proteome provides a more holistic view of colorectal cancer (Kang et al., 2012; Zhang et al., 2014; Vasaikar et al., 2019; Li et al., 2020).

## *In vitro* Colorectal Cancer Proteomics

Primary human samples are the most valuable and accurate representations of human cancer, but they are a limited resource. A solution to this problem is the use of cultured models, which offer greater reproducibility and simplicity, and still have proteomes that are representative of primary colorectal cancer (Wang et al., 2017). The most common models for cancer research are cell lines. Cell lines have seen consistent use (Dumont et al., 2016) since the development of the HeLa cell line from cervical cancer in 1951 (Gey et al., 1952). While two-dimensional (2D) cell culture, where the cells are grown as a monolayer adhered to the bottom of the incubation flask, has been the most common use of cell lines, three-dimensional (3D) cell culture of multicellular spheroids is also possible. These cell cultures are called spheroids due to their shape, and for information on methodology, we would recommend a recent review from Chatzinikolaidou (2016). Even more recently, a new *in vitro* model called an organoid has become popular in cancer research. Several reviews on organoids exist (Clevers, 2016; Drost and Clevers, 2018; Xu et al., 2018), but their potential for proteomics is still relatively unexplored. In this section, we will review recent colorectal cancer proteomics research for all three *in vitro* models and discuss the inverse relationship between simplicity and proteomic accuracy as seen between them.

## 2D Cell Culture

Over the years, 2D cell culture has provided a simple, cost-effective model for cancer research and many cell lines have been developed. Importantly, 2D cell culture can be used to answer fundamental questions about cancer cells. Nusinow et al. (2020) recently used quantitative proteomics to screen the cell lines in the Cancer Cell Line Encyclopedia (CCLE). The CCLE is a large-scale database of over 1,000 cancer cell lines and includes information regarding gene expression, genome sequencing, metabolite profiling, drug sensitivity screens, and targeted protein quantification. The CCLE did not have deep proteome profiling until this study. In addition, similar to primary tumor samples, colorectal proteomics using 2D cell culture has recently focused on utilizing a comparison discovery-based approach to identifying protein biomarkers. Tan et al. (2012) used the colon cancer cell line, HCT-116, to identify Stathmin-1 as a protein biomarker of cancer cell migration. Ala et al. (2020) identified the pro-oncogenic role of ERSP1 in the colorectal cell lines HCA24 and COLO320DM. Hu et al. (2018) used HCT-116 and RKO, selectively cultured to be highly invasive, to identify the Cdc42-Cdc42BPA signaling pathway as a marker for colon cancer invasion. Ludvigsen et al. (2020) compared a normal derived colon mucosa cell line (NCM460) and a colon cancer cell line (HCT116) to identify protein biomarkers with increased expression in colorectal cancer, including retinol-binding protein 1. 2D cell culture has also been used to compare non-metastatic (SW480) and metastatic colon cancer cell lines (SW620) (Zhao et al., 2007; Schunter et al., 2017; Torres et al., 2018). These two cell lines derive from the same patient (Stage II for SW480 and Stage III for SW620). Although these two cell lines form poor spheroids (Stadler et al., 2018), they can provide valuable insight into proteins that may determine whether a cancer will metastasize.

One feature of cell lines not available for primary tumor samples is *in vitro* manipulation. These manipulations include dosing with anticancer drugs (Bauer et al., 2012; Valdes et al., 2017; Raimondo et al., 2018; Schroll et al., 2018), inducing autophagy using hypoxia (Lai et al., 2016), and binding growth inhibitors (Michalak et al., 2016), followed by downstream proteomics, an analysis that looks for differences in the proteome after the manipulation. Fanayan et al. (2013) took a different approach and performed a bottom-up proteomics experiment using chromosome-based data analysis. The proteomic data were organized into clusters based on the MS-based data and the chromosomal location of genes. One cluster identified with this method included several tumor suppressor proteins, including caveolin-1 and caveolin-2, associated with a region on chromosome 7 often deleted in the pathogenesis of colorectal cancer.

As seen above, there are many aspects of 2D cell culture that make them desirable for colorectal cancer proteomics. However, due to a lack of 3D structure, vasculature, and other *in vivo* tumor properties, information obtained from 2D cell culture is incomplete. With the introduction of 3D spheroids and organoids, their ability to provide a more biologically accurate environment (reviewed by Yamada and Cukierman, 2007) helps to fill this gap in the knowledgebase. Furthermore, most of the *in vitro* manipulations performed above can also be used for these models as well. Although 2D cell culture will never become obsolete due to its ease and simplicity, 3D cell cultures have increasingly become more common as models for colorectal cancer.

## 3D Cell Culture

Since 2D cell cultures do not have the same structural features as tumors, there is an inherent loss of spatial information and the proteome will not be truly representative of a tumor. With the introduction of spheroids, the cellular environments of tumors are more accurately replicated *in vitro*, while still providing many of the same advantages of 2D cell culture, for instance, ease of use, versatility, and lower experimental cost. Alternatively, a shared disadvantage between 2D cell culture and spheroids is the presence of only cancer cells, so information on the interaction between healthy and cancerous cells that would be present *in vivo* is lost. Another *in vitro* model, organoids, can be grown to contain both populations, solving this issue and providing a more accurate mimetic (Johnson et al., 2020). This section will review recent proteomic literature for both spheroids and organoids.

### Spheroids

The first proteomics investigation of spheroids compared the spheroids’ proteome with their 2D counterparts. Yue et al. (2016) compared both the proteome and phosphoproteome between spheroids and 2D cell cultures of HT29, a colon cell line. Observation of reproducible changes in abundance for several proteins between the populations suggested that spheroids may provide more accurate proteomic information compared to 2D monolayer cultures. Certain proteins and pathways that showed a change in the spheroids were associated with slower growth and decreased cell and DNA replication, mimicking *in vivo* conditions. In a subsequent study, Kim et al. (2018) compared the proteomes of 3D and 2D SW480 cell cultures after the addition of an enzyme inhibitor. Another colon cell line frequently used for spheroids is HCT-116, which Feist et al. (2015) used to detect over 1350 proteins with very little variation between the replicate spheroids. McMahon et al. (2012) characterized the differences in the proteome between the three cellular populations in spheroids, the proliferating outer layer, the senescent middle layer, and the necrotic core, for the HT29 cell line. These cellular populations are also present in *in vivo* conditions, making this study particularly valuable for comparisons between *in vivo* and *in vitro* models. In addition to spheroids grown using immortalized cell lines, Rajcevic et al. (2014) used patient tumor material to produce spheroids with more *in vivo* tissue characteristics than cell line-based spheroids.

Recent studies in our laboratory have used spheroids to test chemotherapy drugs and the effects of glucose. LaBonia et al. (2018) tested the proteomic changes that occur with treatment of FOLFIRI (folinic acid, 5-fluorouracil, and irinotecan), a combination chemotherapy, on HCT-116 spheroids and observed that folinic acid penetrated into the core of the spheroids and several cancer-associated protein pathways were enriched. Schroll et al. (2016) showed that restricting glucose or serum from the growing spheroids resulted in similar proteomic changes. The effect of combining glucose restriction with chemotherapies has also been examined using spheroids (Schroll et al., 2017, 2018). Feist et al. (2017) used spheroids to evaluate differences in histone post-translational modifications and the effect of an epigenetic drug that targets these modifications. The use of spheroids in the proteomics studies above provides important information about how a treatment might affect an *in vivo* tumor, without testing the conditions on actual patients. These data are the first step toward verifying possible treatments for cancer that could be explored in clinical studies.

### Organoids

Organoids are a relatively recent development in comparison to the other models discussed in this minireview. Xu et al. (2018) define organoids as “3D constructs” that can be developed from “embryonic stem cells, induced pluripotent stem cells, somatic cells, and cancer cells.” The use of stem cells is especially important for this technology, in that these cells can be used to make a variety of organoids, including intestinal organoids. Colorectal tumor organoids have very high potential for screening and thus the possibility for the development of organoid libraries has been explored (van de Wetering et al., 2015; Fujii et al., 2016). Another benefit to organoids is that non-cancerous organoids can be grown, so comparisons between healthy and cancer proteomes can be made, something not possible for 2D cell culture and spheroids.

One major disadvantage for organoid research is the use of a substance called Matrigel, an extracellular matrix necessary for preparing and embedding organoids. Unfortunately, Matrigel contains a variety of growth factor proteins that cause severe ion suppression (Abe et al., 2018). In their research, Abe and his colleagues performed phosphoproteomics analysis on colorectal cancer spheroids and organoids embedded in Matrigel. To avoid the ion suppression effects, they incorporated an acetone precipitation of the digested peptides in their workflow, which increased the percentage of the MS/MS spectra associated with peptides from 8.8 to 26.9%.

Gonneaud et al. (2017) wrote an excellent review of mass spectrometry-based proteomics using colon organoids. Bozzi et al. (2017) identified the MIF/CD74 axis as a target for therapeutics in colorectal cancer, in that its inhibition caused cancer cells to be vulnerable to oxidative stress-induced death. More recently, Lindeboom et al. (2018) used a multi-omics (metabolomics, proteomics, transcriptomics, epigenomics, and genomics) analysis to obtain a complete intramolecular view of intestinal organoids. Michels et al. (2019) used proteomic and transcriptomic profiling of colon organoids to observe physiologic and oncogenic responses to Wnt signaling, such as the up-regulated proteins HMGCS2 and CEMIP. Noticeably, proteomics data from colorectal organoids have been limited in the last couple of years, opening the door for novel developments going forward.

## Future Directions/Conclusion

In this review, the various sample types used for recent colorectal cancer proteomics are described. *In vivo* and *in vitro* samples offer different advantages for research, and within the *in vitro* samples, differing degrees of complexity are also present. After reviewing the recent literature, the authors offer their thoughts on the future of colorectal cancer research. We believe that although primary tumor samples will continue to be valuable research tools, the application of proteomics to spheroids and organoids will continue to see increased representation as cell culture technology progresses and proteomics methodologies improve. These *in vitro* models demonstrate greater statistical reproducibility, due to the lack of tumor heterogeneity, and still maintain a relatively representative proteome. In addition, as these models are grown, they are more readily available than tumor samples that need to be collected from patients. While there are still challenges that need to be overcome, *in vitro* models provide a viable alternative to primary tumor samples, not only for colorectal cancer, but many other cancers as well.

## Author Contributions

PL wrote the manuscript. AH helped revise the manuscript. Both authors contributed to the article and approved the submitted version.

## Conflict of Interest

The authors declare that the research was conducted in the absence of any commercial or financial relationships that could be construed as a potential conflict of interest.
